# Conserved environmental adaptations of stream microbiomes in the hyporheic zone across North America

**DOI:** 10.1186/s40168-025-02236-1

**Published:** 2025-12-05

**Authors:** Tom L. Stach, Jörn Starke, Feriel Bouderka, Till L. V. Bornemann, André R. Soares, Michael J. Wilkins, Amy E. Goldman, James C. Stegen, Mikayla A. Borton, Alexander J. Probst

**Affiliations:** 1 Environmental Metagenomics, Research Center One Health Ruhr of the University Alliance Ruhr, Faculty of Chemistry, University of Duisburg-Essen, Essen, Germany; 2 Centre of Water and Environmental Research (ZWU), University of Duisburg-Essen, Essen, Germany; 3 Department of Soil and Crop Sciences, Colorado State University, Fort Collins, CO USA; 4 Energy and Environment Directorate, Pacific Northwest National Laboratory, Richland, WA USA; 5 Earth and Biological Sciences Directorate, Pacific Northwest National Laboratory, Richland, WA USA; 6 School of the Environment, Washington State University, Pullman, WA USA; 7 Centre of Medical Biotechnology (ZMB), University of Duisburg-Essen, Essen, Germany; 8 DOE Joint Genome Institute, Lawrence Berkeley National Laboratory, Berkeley, CA USA

**Keywords:** Stream, Microbial activity, Hyporheic zone, Anthropogenic stress, Microbiome, Climate change, Temperature

## Abstract

**Background:**

Stream hyporheic zones represent a unique ecosystem at the interface of stream water and surrounding sediments, characterized by high heterogeneity and accelerated biogeochemical activity. These zones—represented by the top sediment layer in this study—are increasingly impacted by anthropogenic stressors and environmental changes at a global scale, directly altering their microbiomes. Despite their importance, the current body of literature lacks a systematic understanding of active nitrogen and sulfur cycling across stream sediment and surface water microbiomes, particularly across geographic locations and in response to environmental factors.

**Results:**

Based on previously published and unpublished datasets, 363 stream metagenomes were combined to build a comprehensive MAG and gene database from stream sediments and surface water including a full-factorial mesocosm experiment which had been deployed to unravel microbial stress response. Metatranscriptomic data from 23 hyporheic sediment samples collected across North America revealed that microbial activity in sediments was distinct from the activity in surface water, contrasting similarly encoded metabolic potential across the two compartments. The expressed energy metabolism of the hyporheic zone was characterized by increased cycling of sulfur and nitrogen compounds, governed by *Nitrospirota* and *Desulfobacterota* lineages. While core metabolic functions like energy conservation were conserved across sediments, temperature and stream order change resulted in differential expression of stress response genes previously observed in mesocosm studies.

**Conclusions:**

The hyporheic zone is a microbial hotspot in stream ecosystems, surpassing the activity of overlaying riverine surface waters. Metabolic activity in the form of sulfur and nitrogen cycling in hyporheic sediments is governed by multiple taxa interacting through metabolic handoffs. Despite the spatial heterogeneity of streams, the hyporheic sediment microbiome encodes and expresses conserved stress responses to anthropogenic stressors, *e.g.*, temperature, in streams of separate continents. The high number of uncharacterized differentially expressed genes as a response to tested stressors is a call-to-action to deepen the study of stream systems.

Video Abstract

**Supplementary Information:**

The online version contains supplementary material available at 10.1186/s40168-025-02236-1.

## Background

Streams connect habitats and ecosystems [1–3], scaling from small creeks and brooks often originating in mountains, over mid-sized upland streams to wide lowland rivers which flow into the sea [4, 5]. The community of microorganisms living within the stream are shaped by the distinct geomorphological and hydrodynamic features along the flow path [6, 7]. In turn, stream microorganisms perform important ecosystem services, in surface water and underlying sediments [8, 9]. A systematic approach to characterize streams is presented by the stream order metric also known as the Horton-Strahler number starting at the spring of a stream with 1 and increasing by the joining of streams with the same or lower order [10, 11]. While brooks originating from glaciers or springs show constant temperatures [12], succeeding headwater streams with low stream orders are often characterized by high variations of flow velocity, discharge, and temperature, and large amounts of terrestrial input like leaf litter [3, 13, 14]. Contrastingly, streams classified as higher stream orders are generally wider and faced with less variation compared to low order streams, carry more nutrients and shift towards an autotrophy-driven system according to the River Continuum Concept (RCC) [3]. The change from headwater stream to the mouth is reflected in a change of fish communities [15, 16], and both eukaryotic microbes [9] and prokaryotic microorganisms [17, 18]. Corresponding changes in microbial community functions were shown to be related to the surrounding land use and cover, with differences for streams in wetlands, forests, and agriculture, respectively [17, 19]. Along the stream continuum, organisms living in streams are exposed to natural variations like temperature, light, and input of allochthonous organic matter depending on a daily and seasonal timescale. Over centuries of exposure to these ever-changing factors, stream communities adapted to this situation [20, 21].

Apart from natural variability in environmental factors, anthropogenic influence in the form of stressors on streams also varies within the water column, both in type and intensity [22–25]. In Europe, only 37% of freshwater ecosystems are in a good or high ecological status including streams of all orders [26]. For example, artificial hydromorphological adaptations in the form of channels or dams are prominent stressors for streams at a global scale [27, 28]. Anthropogenic pressures like inflow of wastewater can be concentrated around densely urbanized areas or human activities like mining can lead to inflow of nutrients and increased temperatures at industry sites [29, 30]. By contrast, temperature increase due to human-caused climate change affects streams across all orders, potentially even more glacier-fed streams in mountain areas [31, 32].

Natural and anthropogenic stress influence the ecosystem services provided by the organisms living in the surface water and sediment of streams [33, 34]. However, an exhaustive study covering 1,851 sites in rivers and streams across North America found that healthy biological communities which are essential for provision of ecosystem services were only found in 28% of investigated stream miles between 2018 and 2019 [35]. In particular, the interface of surface water and groundwater, referred to as the hyporheic zone, represents a biological hotspot [36] that is easily prone to stressors [37, 38]. Yet, microbial communities in the hyporheic zone are involved in complex nutrient cycling and actively shape the ecosystem structure [36, 39–42]. Stream sediments contain much higher number of microbes compared to the overlying surface water column [43], and microbiomes living in the hyporheic zone are not directly compositionally comparable to those of the overlying surface water column as demonstrated in, *e.g*., glacier-fed streams [44, 45].

Multiple approaches can be utilized to study the effect of stressors and environmental variables on the interplay of stream microbial biodiversity and activity, such as the deployment of large-scale outdoor mesocosm experiments simulating stressors [46–48], or systematic field sampling of streams of different orders and habitat types [18, 19, 49, 50]. The Worldwide Hydrobiogeochemistry Observation Network for Dynamic River Systems (WHONDRS) represents such a field study with 97 streams sampled in the summer of 2019 sampling study [51], leading to majority of sampling in the Genome Resolved Open Watersheds database (GROWdb) [19]. GROW revealed that the surface water microbiome of streams is characterized by aerobic and phototrophic metabolisms and follows the RCC by changing along the river gradient. However, this study also revealed the impact of environmental factors like stream order or land use on microbial assembly and activity [19]. Yet, apart from selected studies like GROW, genome-resolved activity data from metatranscriptomics analyses remains limited, as many studies either fail to recover metagenome-assembled genomes (MAGs), obscuring individual microbial contributions, or do not incorporate metatranscriptomics, masking transcriptional activities [17, 18, 52–54].

The full-factorial ExStream system which has been applied for organisms of multiple trophic levels in streams worldwide is an example of targeted stressor testing by manipulating abiotic factors [46, 47, 55–59]. In studies based on this mesocosm system and focusing on the microbiome of streams, the complex interaction of multiple anthropogenic stressors has been shown based on both metagenomic and metatranscriptomic analyses, especially for temperature and hydromorphological changes [33, 58, 59]. Both approaches, *i.e.,* field studies and mesocosms, have advantages and drawbacks. For example, field studies of multiple stream sites typically include a multitude of environmental variables which affect the microbiome and also enable extrapolation of obtained results to other catchments. Yet, the interplay of abiotic and biotic factors in natural systems may be too complicated for clearly linking community response to stressors. On the other hand, mesocosm studies offer the possibility to reduce complexity compared to field studies and increase statistical power due to replication. Yet, these advantages stand in contrast to limited generalizability by mesocosms located at single streams. Both approaches, field studies of multiple sites and replicated mesocosms, benefit from a high number of samples, whose combination helps to overcome low assembly quality of individual samples due to high complexity of microbial communities and to recover high-quality metagenome-assembled genomes (MAGs) [19, 59–61].

Here, we overcome obstacles such as limited generalizability from single experiments and hampered recovery of microbial diversity from single samples, by combining hundreds of metagenomes from both field sampling and a *ExStream* mesocosm experiment conducted in Germany in 2023 [59] to generate two comprehensive river databases, one for MAGs and one for genes. By mapping metatranscriptomic and metagenomic reads obtained from 13 stream samples with data on both hyporheic sediment and surface water to our databases, we show that metabolic activity is not always consistent with encoded potential. For example, hyporheic sediments showed increased activity compared to the overlying surface water despite presenting similarly encoded metabolic potential. Additionally, we projected previously identified stress responses in mesocosm experiments to a diverse set of river sediment metatranscriptomes (*n* = 23). This revealed a consistent active stress response across streams in North America.

## Methods

### Study design and aim

This study was designed with the aim of deciphering the relationship between microbial activity of hyporheic zone sediments and environmental factors like temperature, while identifying and characterizing key microbial players governing biogeochemical cycling. To this end, we combined previously published [19, 54, 59] and unpublished data from 363 stream metagenomes building a comprehensive MAG and gene database to establish GROWdb version 2 (GROWdbv2, methods below). We coupled GROWdbv2 to sediment (*n* = 23) and surface water metatranscriptomic data (*n* = 13) to identify distinct metabolic patterns in these two connected ecosystems. Additionally, sediment metatranscriptomes were used to infer the response of the microbiome to the environmental factors by testing differential gene expression (DGE) for temperature (8.5 to 31.4 °C at time of sampling), sediment composition (16.7% to 94.1% total sand), and stream orders (1 to 8), comparing the response derived from microcosm experiments in ExStream which clearly constrained stress responses of individual biomes [33, 58, 59, 62].

### Collection of river sediment and surface water samples

Sampling and processing of sediment and surface water of global rivers samples has been conducted within the project Worldwide Hydrobiogeochemistry Observation Network for Dynamic River Systems (WHONDRS, https://www.pnnl.gov/projects/WHONDRS) [51], with surface water data previously reported [19]. Sampling followed the protocol for the summer 2019 sampling study [63] and was performed in August 2019. Briefly, sediment was taken within a 1 m^2^ area from the streambed (1–3 cm depth) at five to ten locations per stream and preserved in RNALater. Thus, all sediments samples analyzed in this study target the top layer of the hyporheic zone. Surface water (approximately 1 L) was filtered through 0.22 μm sterivex filters (EMD Millipore), which were capped and filled with 3 mL RNALater. Samples were stored on wet/blue ice or in a 4 °C refrigerator until shipped to Pacific Northwest National Laboratory (PNNL, USA), where samples were subsampled and frozen at −20 °C.

### Nucleic acid extraction and sequencing

Surface water DNA extraction and sequencing methods were previously reported [19]. Here, we build upon the previous study by incorporating the paired sediment metagenomic and metatranscriptomic data. Coextraction of DNA and RNA from sediment was done at Colorado State University (Fort Collins, USA) using NucleoBond RNA Soil kit and DNA Set for NucleoBond RNA soil kit coupled with a DNAse digestion step of remaining DNA in RNA extraction and RNA Clean & Concentrator-5 (Zymo Research Cat. # R1013). Metagenomic and metatranscriptomic sequencing was performed at the Joint Genome Institute (JGI) under the umbrella of a Community Science Program (CSP; proposal: 10.46936/10.25585/60001289; award: 505,780). Metagenomic sequencing followed the protocol described in [19]. Briefly, DNA fragmentation and adapter ligation was done using the Nextera XT kit (Illumina) and unique 8 bp dual-index adapters (IDT, custom design). After enrichment, prepared libraries were sequenced on an Illumina NovaSeq sequencer according to a 2 × 150 nucleotide indexed run program. Metatranscriptomic sequencing and library prep was done using JGI established protocols based on rRNA removal (Qiagen FastSelect probe sets for bacterial, yeast, and plant rRNA depletion (Qiagen)), Illumina TruSeq Stranded mRNA Library prep kit (Illumina), and Illumina NovaSeq sequencer following a 2 × 150 nucleotide indexed run recipe. Collectively, in this study we publish 53 metagenomes, analyzing them together with previously published 310 metagenomic datasets to build databases for subsequent analysis of 23 so far unpublished stream metatranscriptomes [19, 45, 54, 59] (Table SI1 and SI3).

### Metagenomic data processing and construction of GROWdbv2 MAGs

We combined multiple existing datasets with the aim of compiling a comprehensive database of metagenome-assembled genomes (MAGs) from river ecosystems around the world (Table SI2). From the new metagenomes (*n* = 53) reported here, we recovered 1,696 new MAGs following protocols established by GROWdb [19]. Briefly, metagenomic reads were trimmed using sickle (v1.33, [64]) and assembled with IDBA-UD (v1.1.0, [65]) or MEGAHIT (v1.2.9 [66],) and resulting contigs subsequently binned with metabat2 (v2.12.1, [67]). In total, our database comprised 6,724 MAGs originating from GROWdb (3,824 previously published MAGs [19] and 1,696 unpublished MAGs), *ExStream* mesocosm experiment (69 MAGs, [59]), Erpe river study (1,033 MAGs, [45]), and Columbia river study (102 MAGs, [54]). Taken together, 5,915 originated from surface water samples, 478 from sediment, and 331 from porewater of sediments. A dereplicated set of medium and high-quality MAGs with > 50% completion and < 10% contamination was obtained by running dRep (v3.5.0, [68]) at 99% identity with CheckM2 quality assessment as input (v1.0.2, [69]).

The resulting 3,741 MAGs, representing GROWdbv2, were taxonomically annotated using the classify_wf workflow of GTDB-Tk (v2.4.0, [70]) with reference data version r220. The concatenated sequence of all genomes was used for gene prediction using prodigal (v2.6.3, [71], -q -f gff -m -p meta) and a mapping index was built using Bowtie2 (v2.4.5, [72]). Metabolic annotation was performed with DRAM (v2beta, [73]) based on predicted genes using the following modules: CAMPER, dbCAN, Genome_stats, KEGG, MEROPS Peptidases, Heme Regulatory Motifs Counts, and Pfam. Genes playing a part in microbial energy metabolism were grouped using the distill sheets (Table SI7) as part of DRAM.

### Metatranscriptomic data processing and microbial expression calculation of MAGs

Metatranscriptomic sequences were quality checked and trimmed following the workflow established by [19]. Briefly, trimming filtering was done using bbduk and rqcfilter2 embedded in BBTools (Bushnell, https://jgi.doe.gov/data-and-tools/bbtools/bb-tools-user-guide/). The microbial fraction of metatranscriptomic reads was estimated using SingleM (v0.18.3, [74]).To assess MAG activity, trimmed and quality-checked metatranscriptomic reads were mapped to the concatenated multi-fasta file of all genomes using Bowtie2 (v2.4.5, -D 10 -R 2 -N 1 -L 22 -i S,0,2.50, [72]), filtered at 97% minimal identity and used to calculate counts of predicted genes with htseq-count (v2.0.5, [75]). Counts were normalized and transformed to geTMM (gene length corrected trimmed mean of M-values) using edgeR package [76].

### Identification and characterization of MAGs active in sediments

Lineage-specific microbial activity in sediments was obtained by investigating MAGs active in those samples. For that, genes were only considered if expressed in at least two out of 26 samples (13 streams with data on both surface water and sediment) and a MAG was counted as active with a minimum of 20 actively expressed genes following [19]. Thresholds of metatranscriptomic analysis were chosen to stay comparable with the first study on GROWdb [19]. Sulfur and nitrogen pathways encoded and expressed in MAGs based on annotations by DRAM were visualized using Affinity Designer (v1.10.8). A phylogenetic tree of active bacterial MAGs was inferred via GTDB-Tk (v2.4.0, [70]) based on the de_novo workflow with “p__Acidobacteriota” as outgroup. Plotting was done in R using packages ggtreeExtra [77] and ggnewscale [78]. In case of homologous genes, *i.e., nxr* vs. *nar* and *amo* vs. *mmo*, identity of genes was verified using phylogenetic analyses. First, sequences were aligned using mafft (v7.407, -auto, [79]). Then, the alignment was trimmed using trimAL (v1.5.rev0, -automated1, [80]) and a tree was built with IQ-TREE (3.0.1, -alrt 1000 -bb 1000 -m C20+G+F, [81]). Accession numbers of reference genes, partially compiled from [82], used herein can be found in Fig. S2 and S3, and alignments with corresponding treefiles in SI8.

### Clustering, annotation, and mapping of genes from metagenomic assemblies

A gene database was built from assemblies of 363 metagenomes (Table SI3) corresponding to those used for the MAG database (see above; assembly methods have been described in the publication from which the data originated and which are cited in Table SI3). All contigs/scaffolds from metagenomic assemblies were filtered to a minimal contig/scaffold length of 1500 base pairs and concatenated. Gene prediction, annotation, mapping of quality-checked metatranscriptomic and metagenomic reads (trimming done as described in [19]), and counting of hits was done as explained above for the MAG analysis. Amino acid sequences were clustered using MMseqs2 at 95% identity [83] and singletons were excluded from further analyses resulting in 11,043,548 clusters. To enhance read recruitment, reads were mapped against all genes for all assemblies. Since this mapping did not solely contain clustered representative sequences, counts were summed by cluster after mapping. This procedure was done for all metatranscriptome samples and metagenomic samples.

### Effect of environmental factors on sediment gene expression

Transformation to geTMMs for resulting gene expression counts was done as explained above with the same thresholds. Based on the 23 metatranscriptomic sediment samples, the influence of abiotic environmental factors on the microbial activity was assessed. Specifically, temperature, water depth, percentage of total sand in sediment, nitrogen and carbon content, and stream order were extracted for the respective sampling points from WHONDRS (Table S1) [63, 84]. Multivariate statistics were done based on the Bray–Curtis Dissimilarity on normalized counts. These included permanova tests (adonis2, permutations = 999, by = ”margin”) and nonmetric multidimensional scalings (NMDS) [85–88]. The richness of active genes per sample and the number of shared active gene clusters between samples was visualized using UpSetR [89]. The count of active genes per sample was correlated using the native R stats package with the prokaryotic fraction in metatranscriptomic reads as inferred by SingleM (v0.18.3, [74]). Likewise, prokaryotic fraction in metatranscriptomic reads was also correlated with the temperature of the sample.

Differential gene expression was tested for the three most significant environmental factors as inferred by multivariate statistics, *i.e.,* temperature, percentage of total sand, and stream order. Therefore, the dispersion of gene counts was estimated using estimateDisp function (desig*n* = Temperature + Percent_Tot_Sand + Stream_order, robust = T) and a generalized linear model fitted with glmQLFit embedded in edgeR (v4.0.16; [76]). Significant up- and downregulated genes were identified with an adjusted *p*-value < 0.05 (Benjamin-Hochberg) and an absolute log2-fold change of 0.75. These thresholds were chosen to avoid missing differentially expressed genes due to the lack of replicates, while still maintaining a high threshold for significance (adjusted *p*-value < 0.05). For the respective genes, functions were annotated using DIAMOND blast (v2.0.15; blastp –fast -e 0.00001 -k 1; [90]) against FunTaxDB (v1.4 from Nov. 2023; [91]), based on UniRef100 [92] to align with the workflow used in [59]. Data was plotted using ggplot2 [93].

## Results

**Fig. 1:**
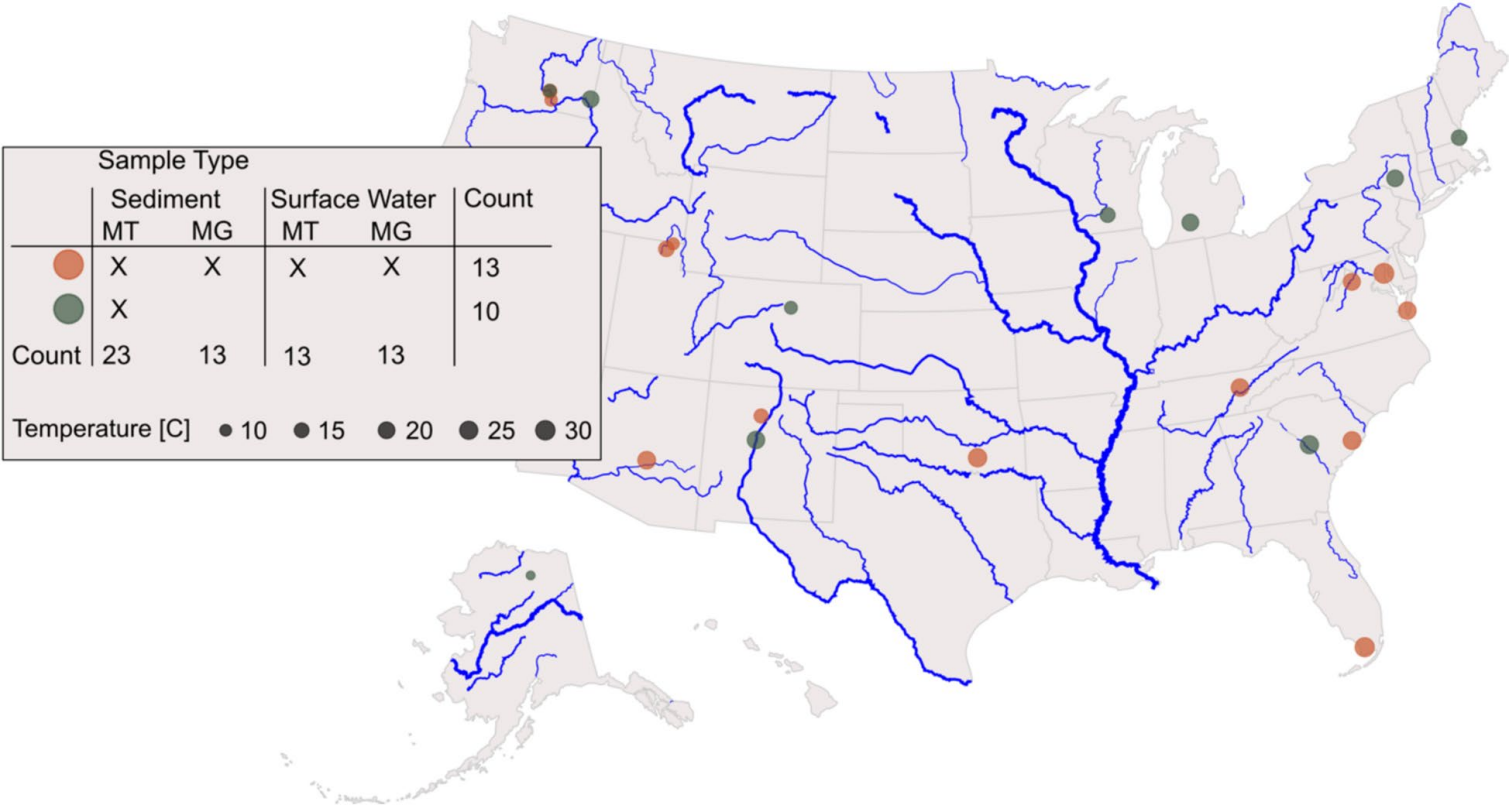
Geographic locations of river samples across North America used in this study. In total, there were 23 samples, 13 of which have metagenomics and metatranscriptomics data from both sediment and the associated water column (orange). Ten of the samples were sediment metatranscriptomes only (green)

To obtain a comprehensive overview of metabolic activity in stream sediments as a function of environmental factors, 23 samples from 20 different streams were surveyed. Environmental factors measured across all sites included surface water temperature that ranged from 8.5 °C to 31.4 °C at the time of sampling, sediment sand composition that varied between 16.7% to 94.1% total sand, and stream orders from 1 to 8 (Fig. 1, Table SI1).

Metatranscriptomes and metagenomes were mapped to two comprehensive databases built for this study. First, 6,724 metagenome assembled genomes (MAGs) were dereplicated at 99% identity to 3,741 medium and high-quality genome representatives forming the baseline for genome-centered analysis. The built gene database contained 11,043,548 clusters (95% AAI) and was used for detailed analysis of stream metabolic functions and microbiomes’ response to environmental stressors.

### Metabolic activity of stream sediments is greater than that of surface water

**Fig. 2:**
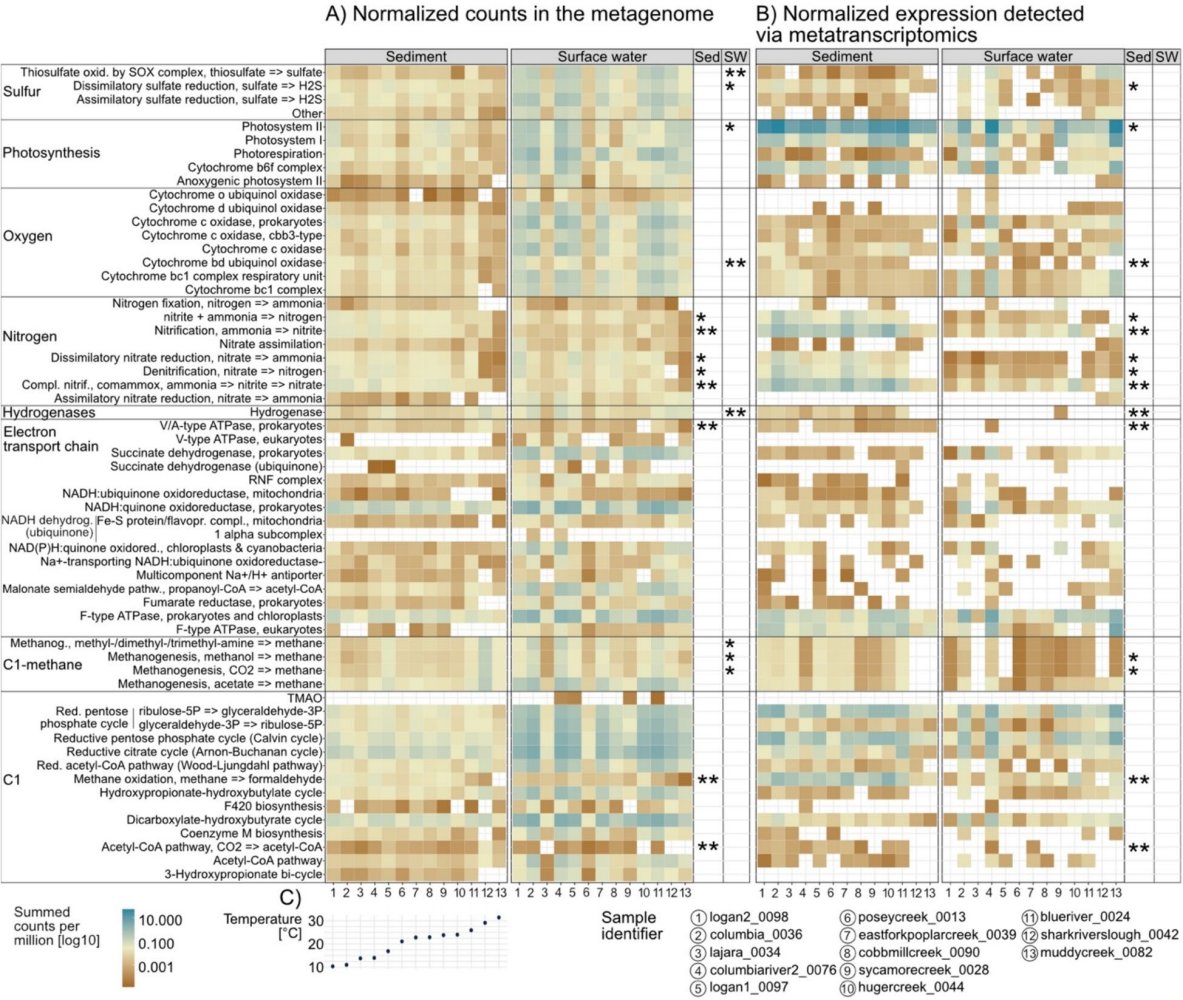
Comparison of microbial energy metabolism in sediment and surface water, encoded in the metagenome (**A**), and its activity measured via metatranscriptomics (**B**). Samples were sorted according to increasing temperature (**C**). The mean counts per pathway were tested for significant differences between surface water and sediment per method (Kruskal–Wallis-Test, *n* = 13, visualized in Fig. S1, for data see Table SI6); a significantly higher mean is marked by significance indices in the respective column (* and ** for adj. *p*-value (BH) < 0.05 and < 0.01, respectively). While in the metagenome selected functions are more abundant in surface waters than sediment samples, expression rates are always greater in the sediment samples compared to surface water, if significant differences were detectable

Genetic information of all microbiomes across the 13 samples encoded for main energy conservation pathways*,* including photosynthesis, aerobic and/or anaerobic respiration, in both sediment and surface water, based on mapping to the comprehensive river gene database built in this study (Fig. 2). While surface water encoded significantly more genes whose products are involved in sulfur (*i.e*., SOX complex and dissimilatory sulfate reduction) and methane cycling, significantly more enzymes for nitrogen (e.g., nitrification or comammox) and other C1 cycling (*i.e*., methanogenesis) were encoded in sediment metagenomes (Kruskal–Wallis-Test, Benjamini-Hochberg-adj. *p*-value < 0.05, see Fig. 2). This finding is in stark contrast with gene expression measured via transcriptomics in this study, *i.e*., no metabolic pathway was detected to be significantly higher expressed in surface water compared to the sediment, only vice versa. As expected, only 20% of pathways recruited reads from all metatranscriptomes as certain genes might be expressed in too small a number to be captured in metatranscriptomics (if expressed at all). For example, “anoxic photosystem II” was encoded in all but one metagenome, but only detected in metatranscriptomes of three out of 13 surface water samples and six out of 13 sediment samples (Fig. 2). Besides the discrepancy between functional potential and measured gene expression, significantly higher expression profiles of pathways (Kruskal–Wallis-Test, Benjamini-Hochberg-adj. *p*-value < 0.05, see Fig. 2) were detected in the sediment samples compared to the surface water. Most striking, pathways that were more frequently encoded in the surface water like methanogenesis (including, *e.g*., key gene, *mcrA*, for methanogenesis), sulfur cycling (including, *e.g*., key gene, *dsrA/B*, for dissimilatory sulfate reduction), or hydrogen oxidation/production displayed significantly higher expression patterns in the sediment samples (Kruskal–Wallis-Test, *p*-value < 0.05, see Fig. 2). Notably, the two sediment samples with the highest temperature during sampling, i.e., shark river slough (“sharkriverslough_0042”, Florida, USA) and muddy creek (“muddycreek_0082”, Maryland, USA), exhibited consistently fewer expressed pathways than those in other sediment samples (only 49% of pathways were expressed in both samples), particularly pathways related to methane metabolism. When excluding these two samples from the analyzed dataset, the difference between surface water and sediment might have appeared even more pronounced, with more pathways showing significantly higher expression in sediment samples.

### Sulfur and nitrogen cycling in stream sediments is a community endeavor

**Fig. 3:**
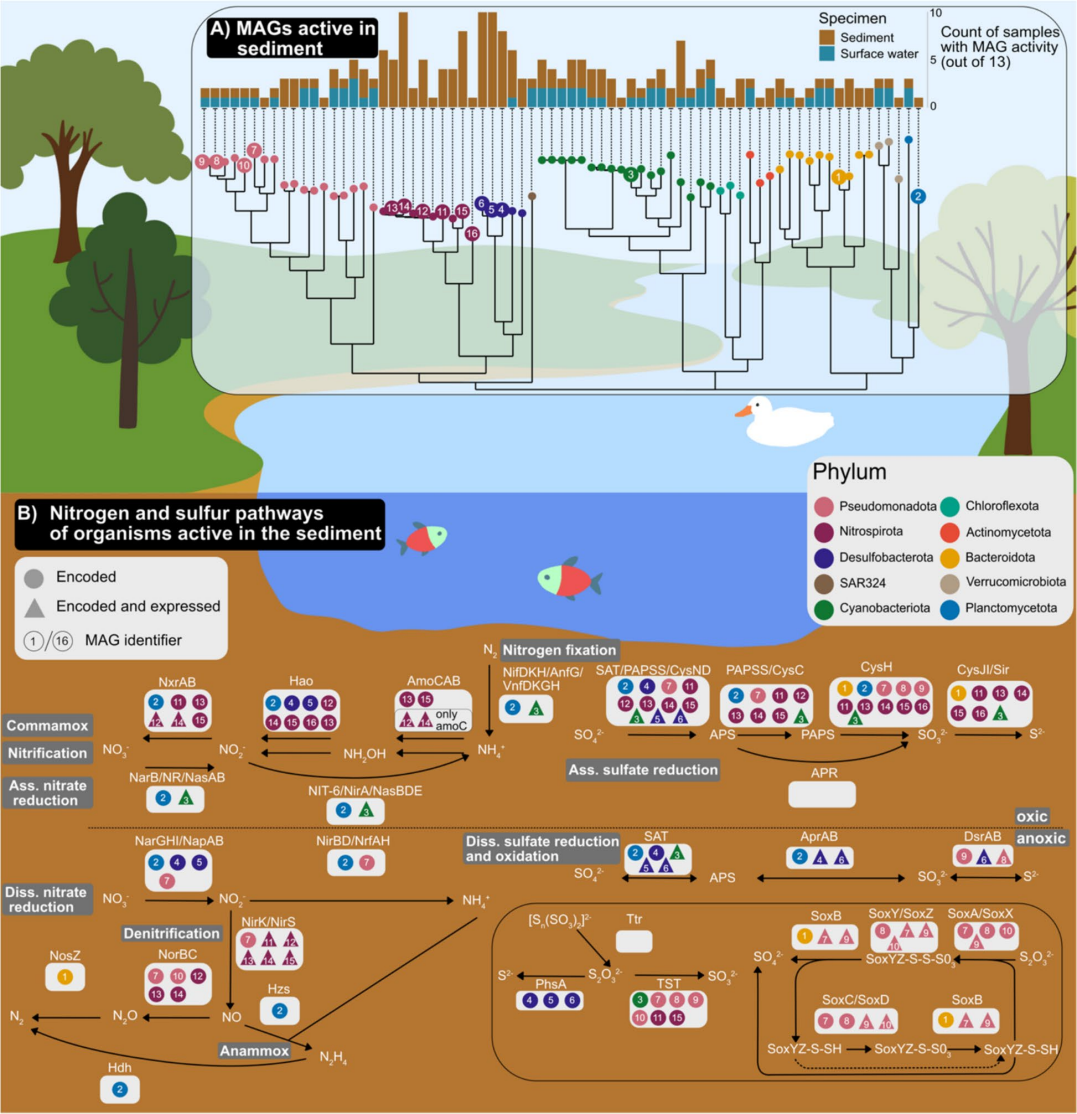
Schematic overview of key metabolic processes governed by MAGs active in the sediment. **A** Phylogenetic tree of MAGs found active in sediments (> = 20 genes expressed) and number of samples with activity of respective MAGs in sediment and water samples, respectively (Table SI2). The MAG identifiers (Tables SI2 and SI9) refer to genomes of organisms active in nitrogen or sulfur cycling (**B**). Genes of these pathways were expressed by organisms which were mostly found to be active in sediments only, especially Nitrospirota and Desulfobacterota. To be assigned as expressed by an organism, genes had to be expressed in at least one sediment sample. In case of reactions involving multiple enzymes, ⅔ of the respective genes had to be encoded or expressed to be included in the figure (data given in Table SI9). For homologues such as *amoA/B/C* and *mmoA/B/C* and *nxrA/B* and *narG/H*, phylogenetic trees were inferred to assign the correct annotation (Figs. S2 and S3)

The analysis of metagenome-assembled genomes (MAGs) based on our river MAG database followed by expression analysis across thirteen samples (Fig. 1), was in agreement with transcriptomics analyses for which we used protein clusters (Fig. 2). For instance, eleven out of 33 organisms which were found to be active in the sediment and not in the water phase were connected to sulfur and nitrogen cycling (Fig. 3). Thus, the MAG-centered analysis was framed for the hyporheic sediment with 75 MAGs found to be active in the sediment and spanning ten bacterial phyla identified as active (Fig. 3A). For example, MAGs belonging to phylum *Nitrospirota* (MAG identifier 13–16) were found to be active by showing gene expression only in sediments and not in the overlying surface water.

A detailed reconstruction of the underlying pathways for both sulfur and nitrogen turnover in sediments revealed the interplay of multiple taxa (represented by MAGs) responsible for the two nutrient cycles. The close phylogenetic relationship between Nitrospirota and *Desulfobacterota* (Fig. 3A) was also reflected by shared possession of genes associated with complete ammonia oxidation (COMMAMOX) and assimilatory sulfate reduction, respectively (Fig. 3B). In detail, Desulfobacterota MAGs contained *Hao* genes, whose products were involved in using hydroxylamine and the production of nitrite or nitrous oxide, respectively [94, 95]. Three Nitrospirota MAGs (MAGs 13–15) affiliated to family *Nitrospiraceae* (one of them genus *Palsa* (MAG 15)) encoded all genes for both pathways that are active under oxic conditions. Key enzymes of the COMMAMOX pathway, *i.e.*, *nxr and amo*, were also expressed by *Nitrospirota* (represented by MAGs 12 and 14). The metabolic versatility of this phylum was also demonstrated by expression of key denitrification genes like *nirK* that encodes for nitrite reductase which catalyses the reduction of nitrite to nitric oxide. Sulfur cycling by *Desulfobacterota* was likely mediated via dissimilatory sulfate reduction (via *dsrAB*, *aprAB*, and *SAT*) in the anoxic part of the sediment. Pseudomonadota encoded and/or expressed key pathways like the *Sox* system for sulfur oxidation or assimilatory sulfate reduction.

Apart from these two main phyla, meaning *Nitrospirota* and *Desulfobacterota*, being responsible for sulfur and nitrogen cycling, two other taxa were active across multiple pathways. As such, the Cyanobacteriota MAG (MAG 3) of family *Nostocaceae* encoded for active nitrogen fixation, assimilatory nitrate reduction, and assimilatory sulfate reduction across samples. Another generalist for sulfur and nitrogen cycling in stream sediments was represented by a MAG annotated as *Candidatus Brocadia* sp., phylum Planctomycetota. This MAG encoded for half of the steps (14 out of 28 genes) of sulfur and nitrogen cycling depicted in Fig. 3, even for both homologs of the nitrate reductase enzyme (*narGH*) and nitrite oxidoreductase (*nxrAB*) (see Fig. S3). Taken together, transcriptomes mapped to our MAG database demonstrated that multiple organisms like *Nitrospirota* were involved in nutrient cycling and deemed specialists as focused on specific pathways. Besides the many specialists, we also identified multiple organisms that were rather generalists, *e.g*., *Candidatus Brocadia* sp., with high metabolic versatility and expressed functions that catalyzed both nitrogen and sulfur cycling (Fig. 3 and Table SI9).

### Microbial activity in the hyporheic zone is influenced by temperature and sediment composition

Although we detected explicit patterns between sediment and water samples, microbial gene expression was not detected to be homogeneous across all sediment samples (Fig. 2), suggesting a meaningful influence of environmental factors in the hyporheic zone. To test this hypothesis, key factors (*e.g.*, being important for streams ubiquitously and/or describe general aspects of streams) were selected from the accompanying WHONDRS metadata and used for statistical testing based on the expression profiles across all 23 sediment metatranscriptomes (Table S1). To comprehensively test for differential gene expression, we compiled an overarching river gene database from 363 samples comprising 11,043,548 representative genes, whose proteins were clustered at 95% amino acid identity. Temperature and percentage of total sand (differentiated from clay and silt) as continuous variables showed significant association with microbial activity (adonis2, *p*-value < 0.05, Table S1).

**Fig. 4:**
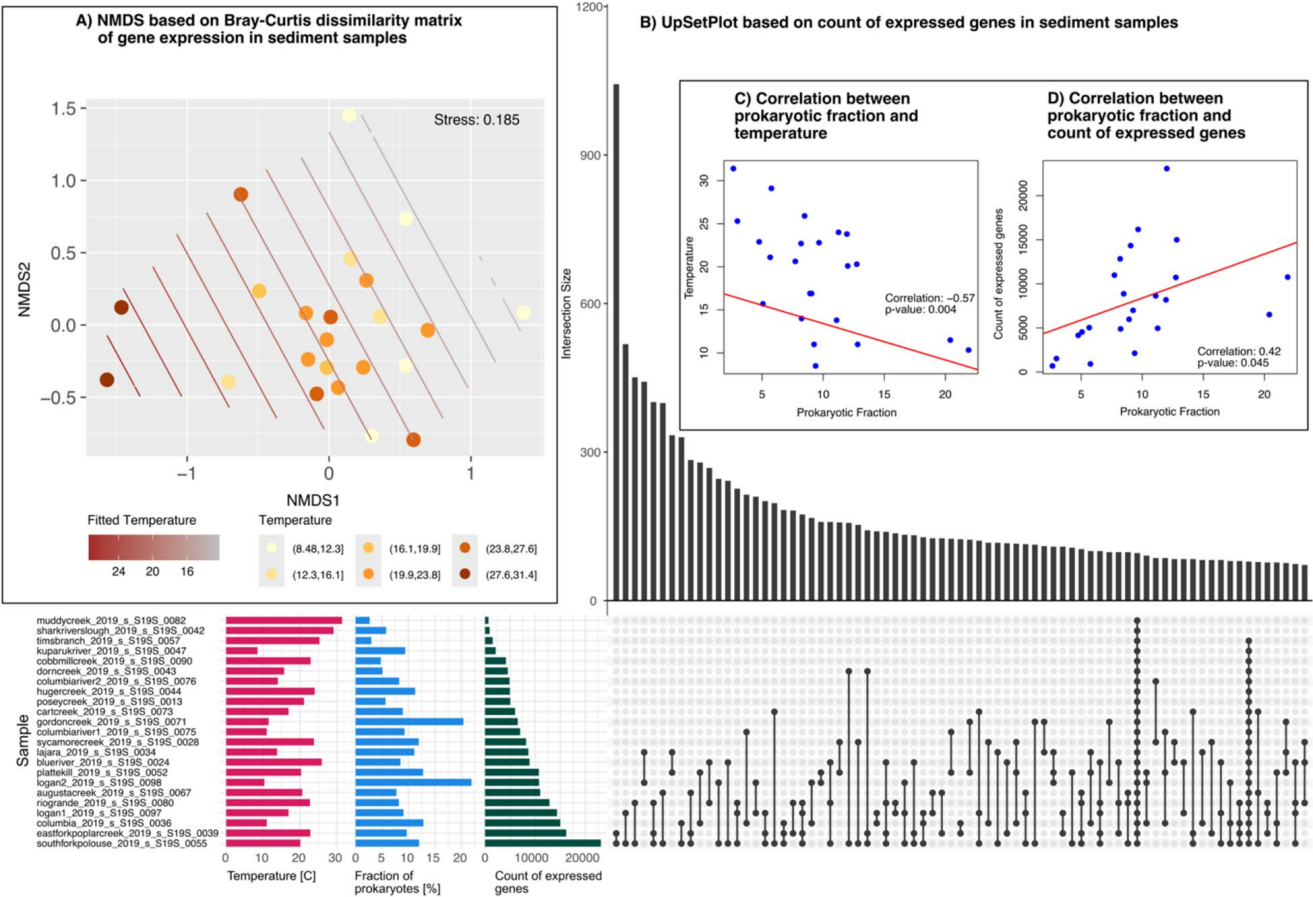
Influence of environmental factors on gene expression in stream sediments. **A** Temperature effect on gene expression depicted by NMDS with fitted temperature and grouping of samples into six temperature intervals. **B** UpSetPlot of active genes (clusters) shared between samples (only top 75 interactions shown, see Table SI4). Samples with the highest temperature have the least expressed genes. **C** and **D** Correlation between prokaryotic fraction and temperature and count of expressed genes, respectively. The prokaryotic fraction of metatranscriptomic reads was inferred with SingleM [74]. There was a significant negative correlation with temperature, and a positive with the count of active genes

Bray–Curtis dissimilarity-based nonmetric multidimensional scaling (NMDS) revealed a clear shift of the microbial expression profile with temperature increase (Fig. 4a), which was accompanied by lower gene expression in samples with higher temperatures, a consequence of location of stream, stream type and climate (Fig. 4b). Correlating the prokaryotic fraction of the microbial activity, as estimated based on metatranscriptomic reads using SingleM [74], with temperature supported the hypothesis that the observed decrease of microbial activity at higher temperatures might be due to an increase of eukaryotic activity (Fig. 4c). An increase of eukaryotic abundance is, in turn, suggested to be negatively correlated with the calculated prokaryotic fraction. Samples with the highest total count of expressed genes shared the most genes (1,043 clusters) with each other. All samples shared 96 actively expressed clusters (Fig. 4b), of which 20 were annotated as Photosystem I/II and 40 as uncharacterized (see Table SI4).

### Temperature activates conserved microbial stress response across stream sediments

**Fig. 5:**
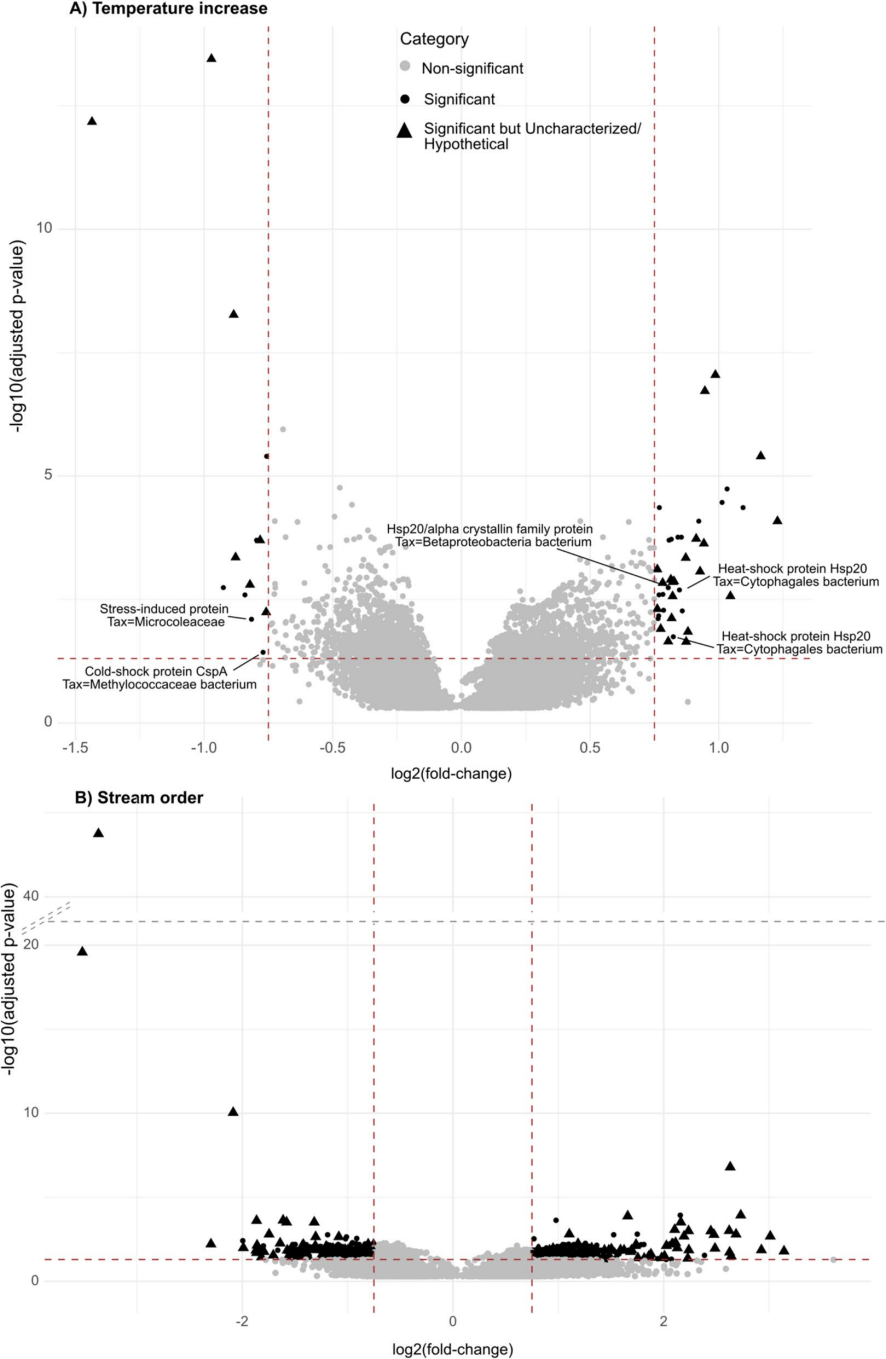
Differential gene expression (adj. *p*-value < 0.05 and abs(LFC) > 0.75) due to temperature and stream order. **A** Temperature increase caused significant upregulation of heat-shock proteins while cold-shock proteins were downregulated (52 differentially expressed genes in total). Annotation was only shown for genes directly related to temperature or stress response. **B** Stream order caused significant up and downregulation of a large set of in total 1,098 genes. Top differentially expressed genes with abs(LFC) > 1 are shown in Supplementary Figs. S4 and S5. A complete list of all genes including functional annotation is given in Table SI5

To infer the specific response of the microbiome to environmental factors, differential gene expression based on the three out of five factors that were identified to have the most significant effect on community gene expression (Table S1), *i.e.,* temperature, percentage of total sand, and stream order (Fig. 5) was performed. Further studies with additional replication would include other factors like depth or nitrogen/carbon percentage.

Significant differential expression (adj. *p*-value < 0.05 and abs(LFC) > 0.75) was detected for temperature (Fig. 5a) and stream order (Fig. 5b), but not for percentage of total sand which is contrary to results from PERMANOVA testing (Table S1). With increasing temperature, stress responses like heat-shock proteins (*e.g.*, Hsp 20), as well as energy-related genes like Photosystem I/II were upregulated (logFC ranging from 0.77 to 1.03 for four genes (adj. *p*-value < 0.05; Table SI5)). By contrast, significant downregulation was detected for cold-shock protein CspA (logFC −0.77 (adj. *p*-value < 0.05; Table SI5)) and a gene annotated as “stress-induced protein” (logFC −0.82 (adj. *p*-value < 0.05; Table SI5)). Compared to the small number of 53 differentially expressed genes by temperature, stream order change led to 1,098 differentially expressed genes, of which 416 up- and 734 downregulated genes were functionally annotated (Fig. 5b, Table SI5). Upregulated genes at higher stream order included seven bacterial genes annotated as heat-shock proteins (Hsp20), other stress response genes like the anti-sigma factor antagonist gene, seven genes encoding cold-shock proteins, or multiple genes regulating translation and transcription or annotated as transposases. Prominent downregulation at high stream order was found by 22 cold-shock proteins. Overall, stress responses were actively transcribed at low stream order and included anti-sigma factors, chaperones, three heat-shock proteins, histones, response regulators, or transcription/translation genes (Fig. 5b, Table SI5).

Focusing on the taxonomic annotation of differentially expressed genes associated with stream order change, 173 belonged to *Nitrospirota*, of which 163 were downregulated with increasing in stream order. Downregulated genes included ribosomal proteins and nitrate oxidoreductase subunits. While downregulated photosystems were annotated with typical freshwater Cyanobacteria, mainly *Chamaesiphon* sp*.*, upregulated photosystems were linked with species also found in marine environments like *Thalassiosira nordenskioeldii.* Similarly, temperature increase also had an effect on gene expression by Cyanobacteria in the form of seven upregulated genes that were taxonomically annotated to genera *Planktothricoides* sp. and one as *Planktotrix.*

In total, 854 functionally annotated genes were found to be differentially expressed (Table SI5) based on changes in either temperature or stream order. A major fraction of these representative genes (216) were identified in the included mesocosm experiment with 64 different metagenomes generated in Germany [59].

## Discussion

Streams represent unique ecosystems with a high spatial and seasonal heterogeneity, not only with respect to the influence of abiotic factors like surrounding land usage and cover, but also to the resulting microbial community living within the surface water [17, 19] and within the sediment and with the hyporheic zone as a hotspot of activity [18, 36]. While already shown for glacier-fed streams [44], we herein establish that the two habitats–surface water and hyporheic zone– exhibit distinct gene expression profiles for multiple key metabolic pathways like nitrogen, sulfur and methane cycling even across different stream orders. Increased expression of genes encoding key metabolic pathways in sediment samples including those with lower than average prokaryotic activity agrees with the concept that describes the hyporheic zone of streams as the ‘river’s liver’ [40]. The finding that surface water metagenomes encode for multiple pathways that are expected in the sediment suggest a suspension of sediment particles in the water column. Yet, active nutrient cycling via microbes employing these pathways seems to be restricted to the sediment. In addition, our analysis of encoded and expressed metabolic functions in stream surface water and sediment suggest that there are conserved metabolic capacities in sediments spanning all stream orders. Active nitrogen and sulfur metabolisms within the sediment microbiomes may also be a microbial response to high levels of anthropogenic land use that introduce such nutrients in the form of fertilizers and storm water runoff (*e.g*., stream Logan, Utah, with sample “logan2_0098”) or wastewater treatment effluents (*e.g.*, stream East Fork Poplar Creek, Tennessee, with sample “eastforkpoplarcreek_0039”) [96–98]. 

A comprehensive river genome catalogue compiled from worldwide river metagenomic studies [19, 51, 54, 59] including 3,741 genomes enabled identification of *Nitrospirota* and *Desulfobacterota* as main players in nutrient cycling in stream sediments. Similar to lake ecosystems [99], cycling of sulfur and nitrogen compounds seems to be a community effort shared by multiple lineages. Such interactions between microbial organisms has previously been described as “metabolic handoffs” for groundwater ecosystems [53]. This concept can now be extended to river ecosystems, rendering this a key concept for freshwater sediment microbiomes. One example of a metabolic handoff identified in the hyporheic zone is partial denitrification with *nirK*, found active only in *Nitrospira* sp.. By contrast, other steps of denitrification were found to be encoded in organisms belonging to *Desulfobacterota*, *Pseudomonadota*, and *Planctomycetota* in case of *narGHI/napAB,* and *Bacteroidota* in case of *nosZ*. While the role of *nirK* in Nitrospira, whose organisms participate in the nitrogen cycle mainly via complete ammonia oxidation (COMMAMOX, [100, 101]), is still not completely understood [102], this finding suggests the metabolic potential for reduction of NO_2_^−^ with other organic substrates as electron donors [103] in river sediments. Elimination of NO_2_^−^ in freshwater as a detoxification process is an ecologically important mechanism with, *e.g.*, increasing NO_2_^−^ concentrations during sulfur-based denitrification thereby constraining nitrogen cycling [104, 105]. Beyond nitrite reduction, the high metabolic versatility of sediment *Nitrospirota* also includes assimilatory sulfate reduction encoded in multiple genomes in this study, which has previously been discussed as an alternative electron and energy source in one Nitrospira species, *i.e.*, *N. gracilis* [106]. These results are a call-to-action to further investigate Nitrospira in rivers along with members of Planctomycetes, as one organism whose MAG was annotated as *Ca*. Brocardia sp. also showed high metabolic versatility based on the encoded genes. Taken together, active genes in the anoxic and oxic metabolic pathways of the river sediments suggest activity of organisms that can swiftly adapt to changing conditions of the hyporheic zone and its available nutrients, especially in the upper 5-cm sediment layer.

Based on the five factors tested in this study, water temperature was found to be a main factor shaping microbial activity in the hyporheic zone is in agreement with results from previous mesocosm experiments [59]. As such, we can verify the applicability of mesocosm experiments like *ExStream* [46, 47, 55–59] also for microbes of the hyporheic zone. Elevated stream water temperature has previously been related to an increase of eukaryotic growth in the form of algal blooms [107, 108], which explains our correlation of fewer gene transcripts with decreases in prokaryotic signals. Comparing our findings with those from mesocosms in Germany [59], we identified a uniform upregulation of temperature-related stress response (*e.g.*, heat-shock protein Hsp20) for microbial communities living in the hyporheic zone across catchments of distinct continents. This uniform stress response suggests that organisms encoding such stress responses have an advantage at the cost of higher energy demand in contrast to more sensitive species during ecosystem degradation caused by anthropogenic stressors. The Asymmetric Response Concept [109], a conceptual framework for stressor effects on stream communities, postulates that species tolerances against stressors are of higher importance in phases of stress impact compared to phases of recovery. While this asymmetry has already been tested and partially verified by mesocosm experiments [59], this study extends the importance of stressor tolerance encoded and expressed by microbes to natural stream ecosystems.

It has been shown that the matrix of the hyporheic zone can influence the community composition of macroinvertebrates [110]. Based on our results from multivariate statistics this can now also be extended to microbial activity at the community level. Sediment composition is an example for the overall high spatial heterogeneity of the hyporheic zone, as its characteristics (*e.g.,* sand versus silt) influence the water flow and water chemistry in the sediment [111]. Being influenced by the sediment composition, hyporheic water flow can in turn affect the surface water temperature on small spatial scales below 0.25 m^2^ [112]. A detailed insight into how sediment composition and microbial activity are entangled was not obtained by this study based on differential expression analysis. Therefore, we suggest designing a targeted sampling strategy with replicates and different sampling points along a gradient of sediment composition within a single catchment and comparison across catchments. Such an effort may overcome limitations of this study, *e.g*., lack of replicates and low number of analyzed factors, and finally uncover specific responses to other environmental factors like mineralogy, total organic matter, nutrient concentrations, or presence of contaminants which all potentially determine microbiome composition and activity in streams.

The River Continuum Concept (RCC) aims to describe the river ecosystem and its longitudinal connectivity based on physical and biological gradients and has been shown to hold true for the microbial activity in the surface water [3, 19]. With the current study, we can extend this concept to the hyporheic zone. In detail, the high number of upregulated genes at low stream order may be related to higher variations of temperature, flow velocity, and nutrients—that are not originating from anthropogenic activities—in lower stream orders compared to wide rivers of high stream order [3, 13, 14]. In a multifactorial mesocosm study based on the *ExStream* setup [46, 47, 55–59], chaperones were found to be significantly upregulated due to lowered flow velocity [59]. Herein, we show that chaperones were along with other stress responses also related to stream order change. The detected higher expression rate of chaperons in low stream order samples suggests that the microbiomes need to adapt quickly to the rapidly changing environmental conditions of headwater streams compared to more stable high order streams. Thus, it could be hypothesized that anthropogenically induced changes of stream flow velocity could pose a similar stress on the microbes living in natural stream hyporheic zones as found in experimental setups.

Taken together, our results warrant further research investigating hydrologically connected stream networks, from headwater to sea to systematically expand the findings of our study. We would like to emphasize that this study benefited greatly from marrying datasets of different working groups and methodological approaches (field and mesocosm studies). Thus, this study reflects the strength of data sharing based on metadata standards and principles like the Data Reuse Information tag (DRI) [113] and FAIR principles [114]. Systematic follow-up studies which include datasets of previous stream research would also provide the opportunity to further characterize the high number of so-far unannotated genes in stream sediments, which harbor a yet undiscovered microbial diversity urging detailed biochemical and bioinformatic investigation.

## Conclusion

The hyporheic zone of stream sediments represents a unique ecosystem characterized by high spatial heterogeneity and high microbial diversity. By comparing 23 stream samples on the basis of comprehensive MAG and gene databases, we revealed an active core microbial community interacting in cycling of essential nutrients like nitrogen and sulfur compounds. At the community level, microbial activity was shown to be affected by sediment composition, temperature, and stream order with a conserved response to stress in the form of up and downregulation of specific genes. Our findings reveal that North American stream microbiomes face constant pressure to adapt to the everchanging nature of streams despite the unique physical and chemical characteristics of each catchment.

## Supplementary Information

Supplementary Material 1: Stach_et_al_2025_Supplememtary_information: Figure S1: Visualization of the statistical comparison of microbial energy metabolism in sediment and surface water, encoded by the metagenome and expressed by the metatranscriptome. Figure S2: Phylogenetic analysis of homologous genes annotated as methane monooxygenase (mmo) and ammonia monooxygenase (amo), respectively, to decipher the functional annotation of genes retrieved from river metagenomes. Figure S3: Phylogenetic analysis of homologous genes annotated as nitrate reductase (nar) and nitrite oxidoreductase (nxr), respectively, to decipher proper annotation of homologous genes from river ecosystems. Table S1: Statistical testing of environmental impact on gene expression of the microbial community structure in stream sediments. Figure S4 and S5: Significantly upregulated (S4) and downregulated (S5) genes with abs(LFC) > 1 and adj. p-value < 0.05 due to stream order increase.
Supplementary Material 2: Table SI1: Meta information of metatranscriptomic and metagenomic samples used in this study. Table SI2: Information on MAGs analyzed in this study. Table SI3: Metagenomic datasets used for building the gene database. Table SI4: Gene identity of genes displayed in UpSetPlot (Figure 4B). Table SI5: List of up- and downregulated genes displayed in Figure 5. Table SI6: Metagenomic and metatranscriptomic counts of metabolic pathways displayed in Figure 2. Table SI7: DRAM distill classification of energy pathways. SI8: Supplementary data for phylogenetic analysis of homologous genes as displayed in Figures S2 and S3. Table SI9: Data on encoded and expressed nitrogen and sulfur pathways as support for Figure 3.

## Data Availability

All metatranscriptomic and metagenomic raw sequencing datasets used in this study are available in the PRJNA946291 repository. The MAG and gene databases have been deposited at Zenodo (10.5281/zenodo.14748957). Used metagenomic assemblies were cited in the Methods section and crosslinked to the respective project in SI Table 3. All metadata and methods regarding sampling procedure are publicly available on the ESS-DIVE data repository from WHONDRS [47–59]. Code for statistical analysis and figure creation has been deposited at the ProbstLab GitHub page (https://github.com/ProbstLab/publication-associated_scripts). Code for statistical analysis and figure creation has been deposited at the ProbstLab GitHub page (https://github.com/ProbstLab/publication-associated_scripts).
